# Nuclear Distributions of NUP62 and NUP214 Suggest Architectural Diversity and Spatial Patterning among Nuclear Pore Complexes

**DOI:** 10.1371/journal.pone.0036137

**Published:** 2012-04-27

**Authors:** Yayoi Kinoshita, Tamara Kalir, Peter Dottino, D. Stave Kohtz

**Affiliations:** 1 Department of Pathology, Mount Sinai School of Medicine, New York, New York, United States of America; 2 Department of Oncological Sciences, Mount Sinai School of Medicine, New York, New York, United States of America; 3 Obstetrics, Gynecology, and Reproductive Science, Mount Sinai School of Medicine, New York, New York, United States of America; University of Colorado, Boulder, United States of America

## Abstract

The shape of nuclei in many adherent cultured cells approximates an oblate ellipsoid, with contralateral flattened surfaces facing the culture plate or the medium. Observations of cultured cell nuclei from orthogonal perspectives revealed that nucleoporin p62 (NUP62) and nucleoporin 214 (NUP214) are differentially distributed between nuclear pore complexes on the flattened surfaces and peripheral rim of the nucleus. High resolution stimulated emission depletion (STED) immunofluorescence microscopy resolved individual NPCs, and suggested both heterogeneity and microheterogeneity in NUP62 and NUP214 immunolabeling among in NPC populations. Similar to nuclear domains and interphase chromosome territories, architectural diversity and spatial patterning of NPCs may be an intrinsic property of the nucleus that is linked to the functions and organization of underlying chromatin.

## Introduction

Nuclear pore complexes (NPC) assemble in the nuclear envelope (NE) from more than 30 different proteins, and are organized into multiprotein subcomplexes. Multiples of eight units of each subcomplex assemble into the modular structures of the pore, which include a symmetric pair of core inner ring complexes, an asymmetric pair of annular rings on the cytoplasmic and nuclear faces, and asymmetric filamentous structures projecting into the nucleoplasm and cytoplasm [1], [2]. In vertebrates, the conserved NUP107-160 subcomplex forms the essential symmetric pair of inner ring complexes of the pore (reviewed in [3], [4]). During assembly of nascent pores, the NUP107-160 subcomplex is recruited to the nuclear envelope by ELYS/MEL28, a chromatin binding protein [5], and the ELYS-NUP107-160-chromatin complex recruits membrane vesicles containing transmembrane proteins that will anchor the NPC into the nuclear envelope [6]. Each of three transmembrane glycoproteins (POM121, gp210, and NDC1) or combinations thereof can anchor the pore complex in the NE, and their expression varies between cell types and species [7], [8].

Located symmetrically on both the cytoplasmic and nuclear faces of the NPC, NUP155 interacts with Gle1 and nucleoporin pCG1 to mediate mRNA transport [9], [10]. Residing near the nuclear envelope and interacting with lamin B, NUP53 associates with a putative NPC complex containing NUP93, NUP155 and NUP205 [11]. Proteolytic cleavage of a 186 kDa precursor protein results in the generation of NUP98, an FG-repeat containing nucleoporin [12], which resides on both the nuclear and cytoplasmic sides of the NPC [13], and of NUP96, for which evidence has been presented of a role in core inner ring assemble [14]. The NUP62 subcomplex, which contains the FG-repeat nucleoporin NUP62 [15], forms rings on the inner channel of the pore [13], [16]. The cytoplasmic or nuclear annular rings contain eight subunits of NUP88-NUP214 [17], [18], [19] or NUP153 [20], respectively. Filamentous structures projecting into the cytoplasm or nucleus are formed by NUP153/Tpr [20] or NUP358 [21], respectively (reviewed in [4]).

Export of mRNA from the nucleus is a complex process that in yeast and probably in metazoans is linked to mRNA processing. Export proteins that interact with the NPC and mediate transport recognize adaptors that bind maturing RNA, and many of these adaptors are directly involved in RNA processing (reviewed in [22], [23]). Passage of protein cargo through the NPC is mediated by carrier proteins (karyopherins), which include the importins, exportins, and transportins, and differential expression of the karyopherins is an important determinant in commitment to specific cell lineages (reviewed in [24]). Cargo/karyopherin complexes transported from the cytoplasm to the nucleus dissociate in the nucleus by interacting with RanGTP (reviewed in [25]). Likewise, karyopherin- or cargo/karyopherin-RanGTP complexes pass to the cytoplasm, where RanGAP promotes hydrolysis of RanGTP to RanGDP, and dissociation of the complex. RanGDP returns to the nucleus through its interaction with nuclear transport factor 2 (NTF2), and the nucleus guanine nucleotide exchange factor (RanGEF or RCC1) mediates recharging to RanGTP (reviewed in [26]). A shared structural feature of many nucleoporins (referred to as FG-repeat containing) are hydrophobic core amino acid repeats, such as GLFG or FXFG, which mediate selective interactions with carrier proteins involved in nuclear/cytoplasmic translocation. For example, NTF-2 binds primarily FXFG nucleoporins, whereas importin-β and the mRNA export factor TAP bind both FXFG and GLFG nucleoporins [27], [28]. In addition, the functional roles of different FG-repeat proteins are probably not equivalent, and interactions between FG-repeat proteins may influence their activities [29]. Finally, recent studies have shown that some FG-repeat nucleoporins accumulate in the nucleoplasm, and independently of the NPC associate with and function in transcriptional regulation of specific segments of chromatin [30], [31].

The NUP62 complex assembles from O-glycosylated proteins of molecular masses 62, 58, 54, and 45 kDa [15], [16]. The 62 kDa component of the complex, NUP62, contains three domains: N-terminal FG-repeat, central threonine/alanine-rich linker, and C-terminal alpha helical coiled-coil [15]. Similarly, NUP214 contains an N-terminal FG-repeat domain, while the C-terminal region is rich in serine, threonine and proline [17]. Although bound to the ring structure on the cytoplasmic face of the NPC, the FG-repeat domain of NUP214 is flexible and can reside on either side of the pore, and its topology is influenced by the transport state of the NPC or the local concentration of chemical mediators [32], [33]. Nucleoporin 214 has been reported to interact with NUP88/NUP82p [34], NUP358 [35], NUP62 [36], and NUP98 [13]. Although global alterations in the architecture and stoichiometry of the NPCs have been observed after knock-down of NPC components either by genetic or other experimental methods [19], [37], [38], [39], architectural and distributional diversity among native NPC populations in cells are largely unexplored. Here we report that NPCs display architectural and distributional heterogeneity in adherent cultured cells. Populations of NPCs are distinguished by NUP62 and NUP214 immunolabeling and by their spatial distribution on the nuclear envelope.

## Results

### Populations of NPCs distinguished by NUP62 and NUP214 immunolabeling

The three dimensional shape of the nucleus of most adherent cultured cells approximates an oblate ellipsoid, which is essentially a moderately flattened sphere consisting of contralateral surfaces facing either the culture plate or medium connected by an intervening, steeply-arcked rim structure. Several of the microscopic images presented in this report represent stacks of serial optical sections taken from the medium side through the entire nucleus to the culture plate side, and are visualized as a projections of the stack to the x,y, x,z, and z,y planes. By viewing signals from two perspectives, it is possible to distinguish co-localized and disparate immunolabeling. Nucleoporin immunofluorescent signals emanating from the nuclear envelope (contralateral surfaces and rim) are likely to represent NPCs, whereas intranuclear signals, by definition, would not arise from NPCs and may represent nucleoporins involved in functions other than nucleocytoplasmic transport [40].

**Figure 1:**
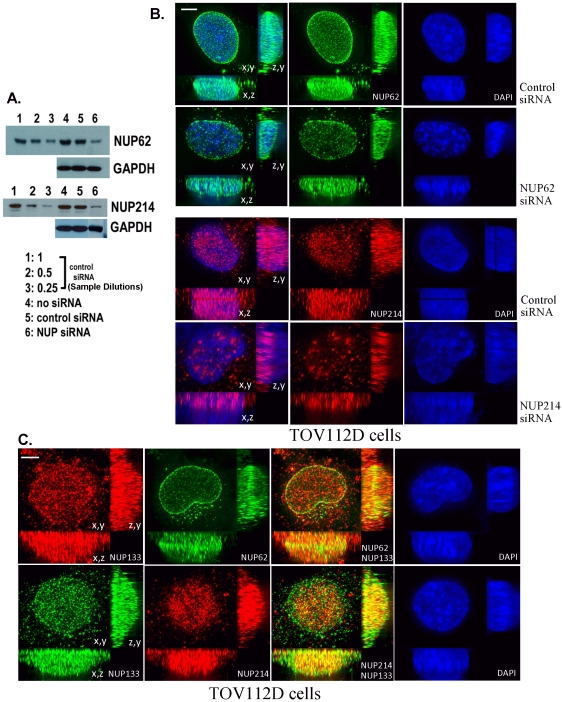
Knockdown of NUP62 or NUP214 in ovarian carcinoma cells (TOV112D) treated with specific siRNAs. A. Constant total protein from siRNA-treated cultures was analyzed for nucleoporin content by immunoblot with the indicated antibodies. Dilutions of the cultures treated with control siRNA are shown for comparison to semi-quantify the extent of knockdown (siRNA knockdown resulted in approximately the same signal intensity as dilution to 25%). B. Immunofluorescence microscopy of TOV112D cells treated with NUP62 or NUP214 siRNAs. Immunofluorescent images represent 3D deconvolved projections of 10–15 um total of optical sections through the z axis. C. Immunofluorescence microscopy of NUP133 and NUP62 or NUP214 in TOV112D cells. Bars represent 2 um.

Western blot analyses revealed that knockdown of NUP62 or NUP214 to ∼25% of control levels resulted from treatment of TOV112D cells (derived from a high grade ovarian carcinoma [41]) with combinations of targeted siRNAs (Fig. 1A). Knockdown of NUP62 was accomplished in 48 hours with a single treatment of three specific siRNAs; knockdown of NUP214 required 72 hours and two treatments with six specific siRNAs. Although knockdown of NUP62 is accompanied by growth inhibition of TOV112D cells, this resulted from mitogenic arrest rather than loss of cell viability [42]. In contrast, NUP214 siRNA treatment of TOV112D cells for extended periods (longer than one week) resulted in death of TOV112D ovarian tumor cells, followed by colonial growth of some remaining cells. For the NUP214 knockdown studies presented below, original NUP214 siRNA treated TOV112D cells were employed and not cells recovered from remaining colonies. Immunofluorescence microscopy revealed that knockdown of NUP62 results in regional loss of immunolabeling among NPCs rather than a general loss of signal from all NPCs (Fig. 1B). In particular, signal is lost from groups of NPCs on the rim of the nucleus (readily apparent when viewing projections on the z,y and x,y planes). Knockdown of NUP214 resulted in loss of signal from the contralateral surfaces of the nucleus, with the remaining signal localized to intranuclear accumulations (Fig. 1B). Intranuclear immunolabeling by the NUP214 antibody is further addressed below, but as this labeling lies distal to the nuclear envelope it would, by definition, not be attributable to NPC-bound NUP214. Double immunofluorescence microscopy of TOV112D cells with NUP62 and NUP133 (a component of the conserved NPC inner core ring complex [38]) revealed co-localization of NUP62 with NUP133 at the nuclear rim; in contrast, double immunofluorescence with NUP214 and NUP133 antibodies revealed preferential co-localization of NUP214 with NUP133 on the contralateral surfaces the nucleus (Fig. 1C).

**Figure 2:**
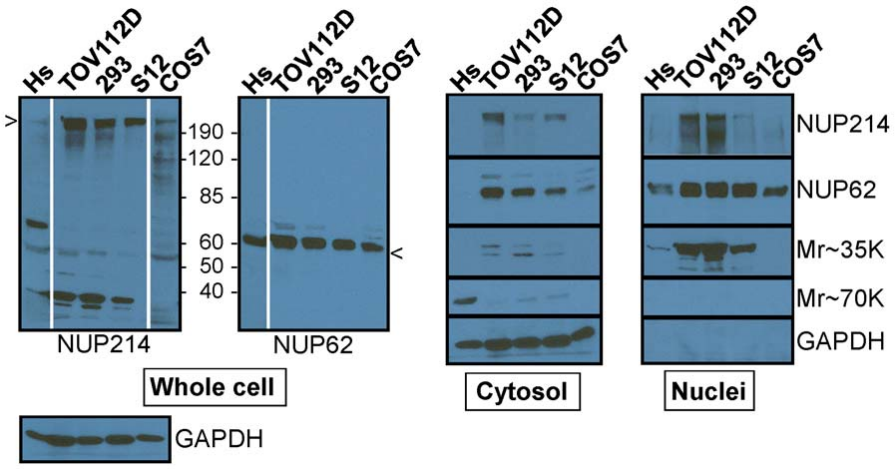
Detection of NUP62 and NUP214 in nuclear and cytoplasmic compartments of cultured cells. Whole cell, cytosolic, and nuclear fractions from Hs 832(C).T (Hs), TOV112D, HEK293 (293), S12, and COS7 cells were analyzed by immunoblot with antibodies to NUP62 and NUP214 (antibodies used were also used for immunofluorescence). Two T75 plates of cells at 80% confluence were used for each preparation (this does not yield equal numbers of cells). One third of the sample was taken for the whole cell extract; the remaining sample was fractionated into cytosol and nuclear components. Extracts were loaded to represent equivalent fractions of starting material. Indicated as Mr∼35K is an additional band observed in the NUP214 immunoblot. The Mr∼70K band present in the Hs832(C).(Hs) whole cell lysate fractionated with the cytosol. The Western blot analyses of the whole cell lysates were completed on single membranes for all five cell lines; however, the exposure times for Hs832(C).(Hs) and Cos 7 cells using the NUP214 antibody and for Hs832(C).(Hs) using the NUP62 antibody were longer. Consequently, those lanes are separated.

Employing a recently reported method that is rapid, gentle, and preserves protein-protein interactions [43], nuclear and cytosolic fractions were isolated from Hs 832(C).T (slowly proliferating fibroblastoid cells derived from an ovarian benign cystadenoma), TOV112D, HEK293(adenovirus transformed cells derived from human embryonic kidney), S12 (a clone derived from SH-SY5Y neuroblastoma cells), and COS7 (green monkey SV40 transformed kidney cells). Samples were immunoblotted with the NUP62 and NUP214 antibodies used for immunofluorescence (Ab1 and Ab4, below). As shown in Fig. 2, binding of the NUP214 antibody was predominantly at the NUP214 band and another band of ∼35 kDa. While the latter band is detected in the nuclear fraction, it does not appear to contribute a signal in immunofluorescence microscopy. For example, while the 35 kDa band is absent from immunoblots of COS7 cell extracts, the NUP214 immunofluorescent patterns of TOV112D (which display the 35 kDa band) and COS7 cells are quite similar (see below). In addition, immunoblots of both HEK293 and TOV112D cells display the 35 kDa band, but their NUP214 immunofluorescent patterns are different. It is likely that the 35 kDa band contains an epitope that is revealed only in immunoblots after denaturation of the proteins in SDS. The NUP62 antibody bound exclusively to ∼62 kDa bands (Fig. 2). The NUP214 and NUP62 antibodies detected varying levels of nucleoporins in the cytosolic fractions, which is consistent with other reports of cytoplasmic stores of nucleoporins in annulate lamellae and other compartments [44], and with immunofluorescence analyses shown below.

### Architecturally distinct NPC populations are positioned on the nuclear envelope and can be distinguished from intranuclear nucleoporins

**Figure 3:**
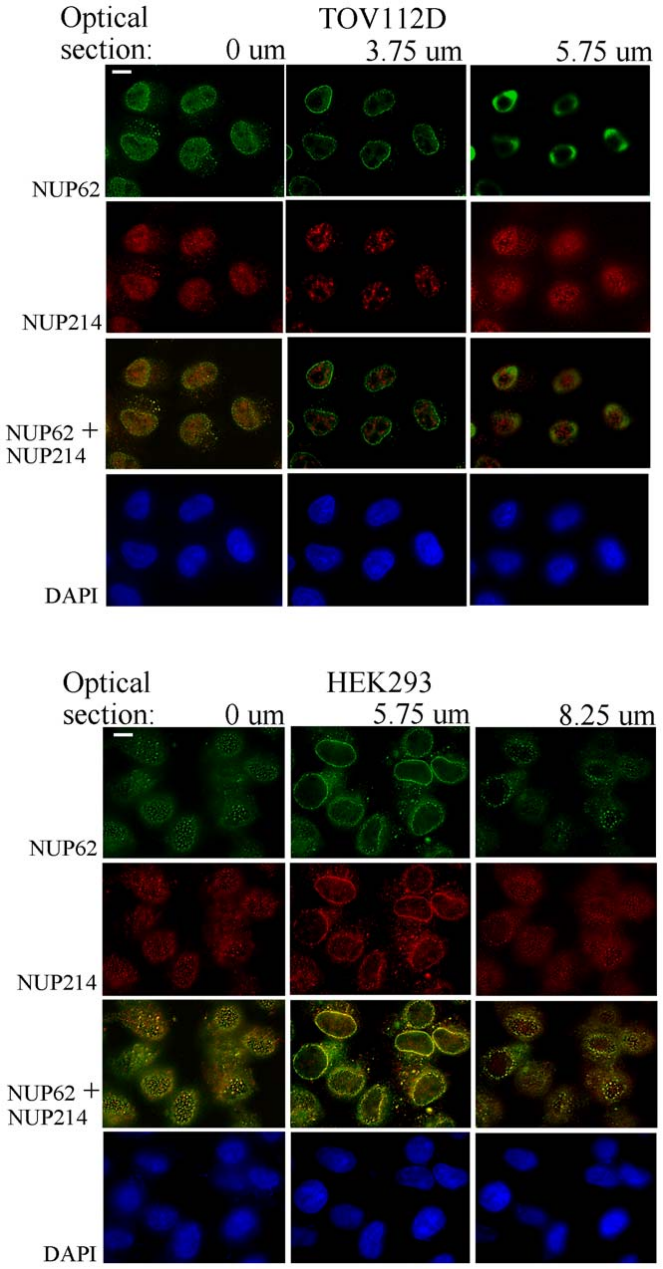
Optical sections reveal NPC populations on contralateral flattened faces and rim of the nucleus. HEK293 and TOV112D cells were immunolabeled with NUP62 (green) and NUP214 (red) antibodies. Optical sections were generated from the culture plate surface upward to the medium at intervals of 250 nm. The first focused nuclear surface plane (0 um), a central plane, and last focused surface plane were selected and 2D deconvolved. Overlay of NUP62 and NUP214 signals is shown, with coincident signals revealed in yellow. Blue, DAPI stain of nuclei. Bar represents 5 um.

To distinguish intranuclear signals from those originating at the nuclear envelope, single optical sections from the nuclear surfaces and the central nuclear plane were observed individually. The TOV112D and HEK293 cell lines were chosen for this analysis because they display predominantly disparate or coincident patterns of NUP62 and NUP214 immunolabeling, respectively (discussed below). In HEK293 cells, optical sections through the central plane of the nucleus display overlapping punctate NUP62 and NUP214 signals on the rim of the nucleus, a distribution considered typical for NPC immunolabeling (Fig. 3). The NUP62 and NUP214 also antibodies co-labeled abundant NPCs on the contralateral surfaces of HEK293 nuclei (Fig. 3). Immunolabeling in the central optical section from TOV112D cell nuclei produced the typical punctate nuclear rim pattern with NUP62 antibody, whereas NUP214 antibody produced dense accumulations of intranuclear labeling and a comparatively weak signal from the nuclear rim (Fig. 3). The NUP214 intranuclear pattern clearly does not represent NPCs as it is neither punctate nor resident on the nuclear surface. Punctate NUP62 and NUP214 immunolabeling observed on the contralateral surfaces of TOV112D cell nuclei displayed only partial overlap.

**Figure 4:**
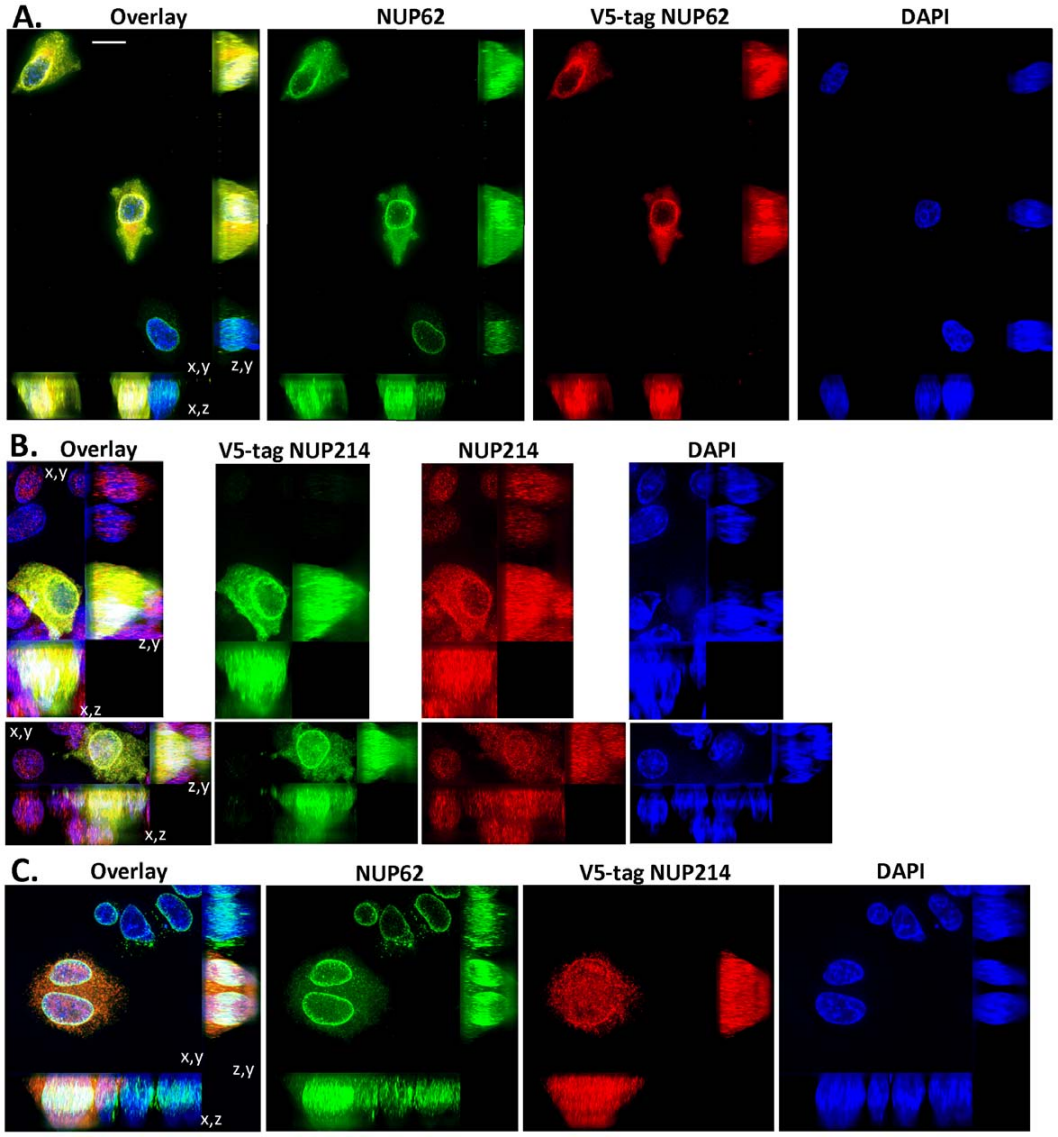
Immunolabeling patterns of native NUP62 and NUP214 antibodies are consistent with those produced by V5 epitope tag antibodies detecting ectopically expressed nucleoporins. Immunofluorescent images represent 3D deconvolved projections of 250 nm optical sections through 10 to 15 um of the z axis. Projections are shown on x,y, x,z, and z,y planes, as indicated. Bar represents 10 um. A. TOV112D cells were transfected with an expression plasmid for V5 epitope-tagged NUP62 and cultured for 72 hours. The distribution of NUP62 was analyzed in pairs of post-mitotic cells expressing V5 epitope-tagged NUP62. Immunofluorescence analyses of NUP62 (green) and V5 epitope-tagged NUP62 (red) were performed with Ab1 and V5 epitope tag antibodies, respectively. Blue, DAPI stain; yellow, overlap between green and red. B. TOV112D cells were transfected with an expression plasmid for V5 epitope-tagged NUP214 and cultured for 72 hours. The distribution of NUP214 was analyzed in pairs of post-mitotic cells expressing V5 epitope-tagged NUP214. The images of the pair of post-mitotic cells shown have been cropped individually to avoid interference from surrounding cells, particularly on the x,z and z,y planes. Immunofluorescence analyses of NUP214 (red) and V5 epitope-tagged NUP214 (green) were performed with Ab4 and V5 epitope tag antibodies, respectively. Blue, DAPI stain; yellow, overlap between green and red. C. TOV112D cells were transfected with an expression plasmid for V5 epitope-tagged NUP214 and cultured for 72 hours. The distributions of NUP62 and NUP214 were analyzed in pairs of post-mitotic cells expressing V5 epitope-tagged NUP214. Immunofluorescence analyses of NUP62 (green) and V5 epitope-tagged NUP214 (red) were performed with Ab1 and V5 epitope tag antibodies, respectively. Blue, DAPI stain; yellow, overlap between green and red.

Further studies of NUP62 and NUP214 immunolabeling in TOV112D cells were performed using expression constructs for V5 epitope-tagged NUP62 or NUP214 cDNAs. Plasmids were transfected into TOV112D cells, and processed for immunofluorescence microscopy 72 hours later using NUP62, NUP214, and/or V5 epitope tag antibodies. Observations were restricted to pairs of post-mitotic cells. As NPCs and the nuclear envelope are disassembled during mitosis then reassembled during abscission and early G1 phase, observation of post-mitotic pairs of transfected cells assures that the V5 epitope-tagged forms of NUP62 and NUP214 have distributed into the total NPC and nucleoporin pools. Cells transfected with V5 epitope-tagged NUP62 or NUP214 expression constructs were analyzed by immunofluorescence with V5 epitope antibody and NUP62 or NUP214 antibodies, respectively. Extensive co-localization of NUP62 or NUP214 immunolabeling with V5 epitope tag immunolabeling was observed in transfected TOV112D cells (Fig. 4A, B), confirming signal specificity of the nucleoporin antibodies. In transfected cells, NUP62 and NUP214 antibody immunolabeling was slightly more extensive than that of the V5 epitope antibody, as the nucleoporin antibodies bind both the transfected V5 epitope-conjugated and endogenous forms of NUP62 and NUP214. As expected, total NUP62 or NUP214 immunolabeling increased in cells transfected with expression constructs (Fig. 4A, B).

**Figure 5:**
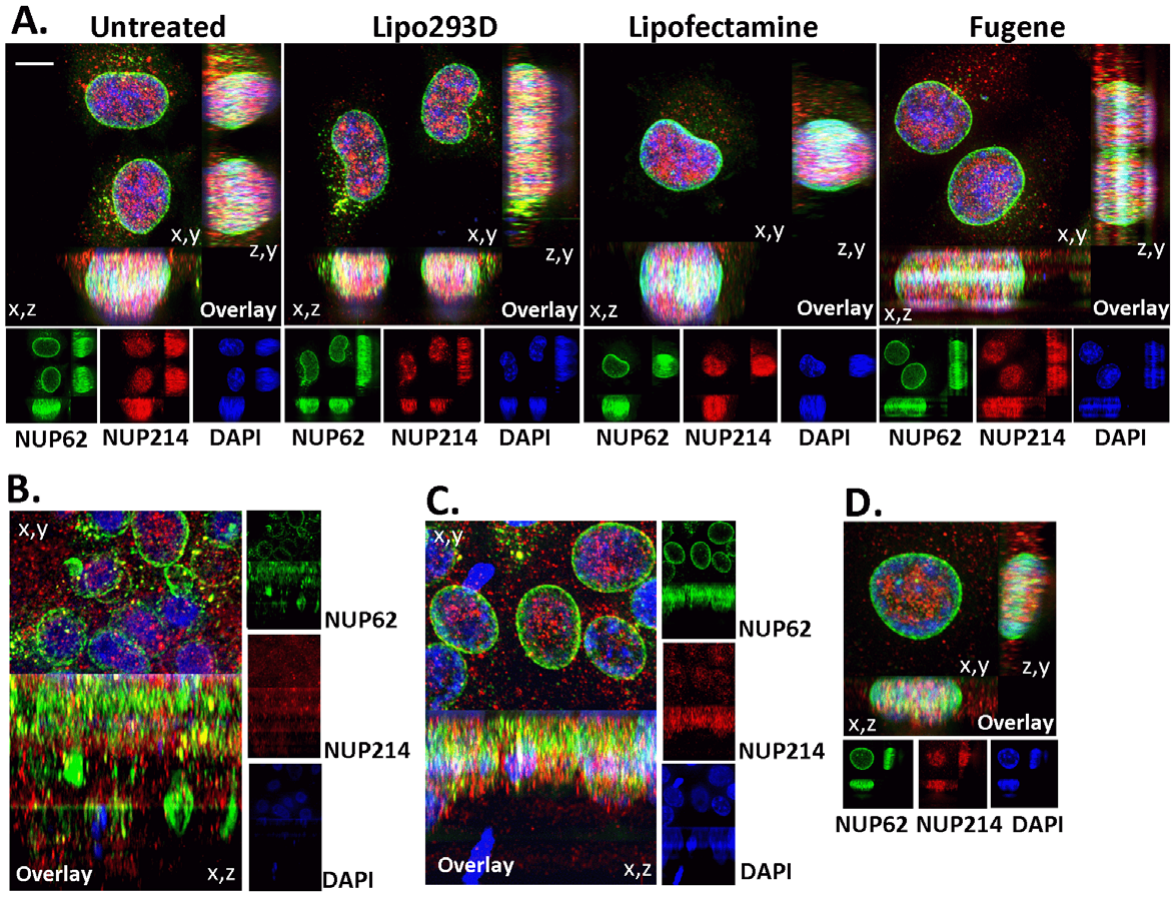
Effects of transfection reagents and cell density on nucleoporin distribution. A. TOV112D cells were treated in the absence of exogenous DNA with Lipo293D (SignaGen Laboratories), Lipofectamine (Invitrogen), or FuGENE HD (Promega), according to the instructions provided by the manufacturers. The cells were processed for immunofluorescence using NUP62 (green) and NUP214 (red) antibodies 48 hours after treatment. Immunofluorescent images represent 3D deconvolved projections of 250 nm optical sections through 10 to 15 um of the z axis. Projections are shown on x,y, x,z, and z,y planes, as indicated. Bar represents 5 um. Blue, DAPI stain; yellow, overlap between green and red. B. TOV112D were cultured at confluence for three days, and analyzed by immunofluorescence microscopy as described above. C. TOV21G cells were cultured at confluence for three days, and analyzed by immunofluorescence microscopy as described above. D. TOV21G cell cultured at low density and analyzed by immunofluorescence microscopy as described above.

The V5 epitope tags were inserted at the N-termini of NUP62 and NUP214. The NUP62 antibody recognizes an epitope within residues 24–178 and the NUP214 antibody recognizes epitopes between residues 1250–1300 of the human form of the protein. The nucleoporin immunolabeling patterns generated by native and epitope tag antibodies overlapped extensively, suggesting that immunofluorescence patterns generated by the native NUP62 or NUP214 antibodies reflect the presence or absence of nucleoporins and not post-translational modifications that alter antibody binding (Fig. 4A, B). The TOV112D cells were transfected with V5 epitope-tagged NUP214 expression construct, and analyzed by immunofluorescence microscopy 72 hours later using NUP62 and V5 epitope tag antibodies. The appearance of nuclei immunolabeled in this manner resembled those in untransfected cells immunolabeled with NUP62 and NUP214 antibodies: intense NUP62 signal near the nuclear rim, and weaker V5 epitope-tagged NUP214 signal at the rim accompanied by common and distinct labeling at the contralateral nuclear surfaces (Fig. 4C). This result supports the differential distributions of NUP62 and NUP214 observed using native NUP214 antibody. The enhanced level of V5 epitope-tagged NUP214 signal observed at the nuclear rim of transfected cells is likely to result from the higher level of total NUP214 expression. Changes in the distribution of NUP214 were observed using Lipo293D (SignaGen Laboratories) transfection reagent in the presence or absence of DNA, but were not observed when other transfection reagents were used, including Lipofectamine (Invitrogen). While Lipo293D is a liposome-based reagent, it contains a proprietary booster peptide that targets liposomal contents to the nucleus and greatly increases transfection efficiency. Accumulation of NUP214 in loci much larger than NPCs was observed after Lipo293D treatment; by observing image stacks from x,y, x,z and z,y perspectives, it is clear that these loci are intranuclear (Fig. 5A). It is intriguing to speculate from this observation that the presence or abundance or certain NPC cargo, such as the nuclear localization peptide present in Lipo293D, can directly influence the nuclear distribution of NUP214. We also asked whether the density of cells influences NUP62 and/or NUP214 immunolabeling patterns. Consistent with their origin in a high grade ovarian carcinoma, TOV112D cells form a poor monolayer with abundant overgrowth and cell death, whereas TOV21G cells (a cell line derived from another ovarian carcinoma; [41]) form a monolayer with an epithelial appearance. In both cell lines, the distributions of NUP62 and NUP214 were consistent between subconfluent and confluent cells (Fig. 5B–D).

### Spatial distribution of architecturally distinct NPCs varies with cell type

**Figure 6:**
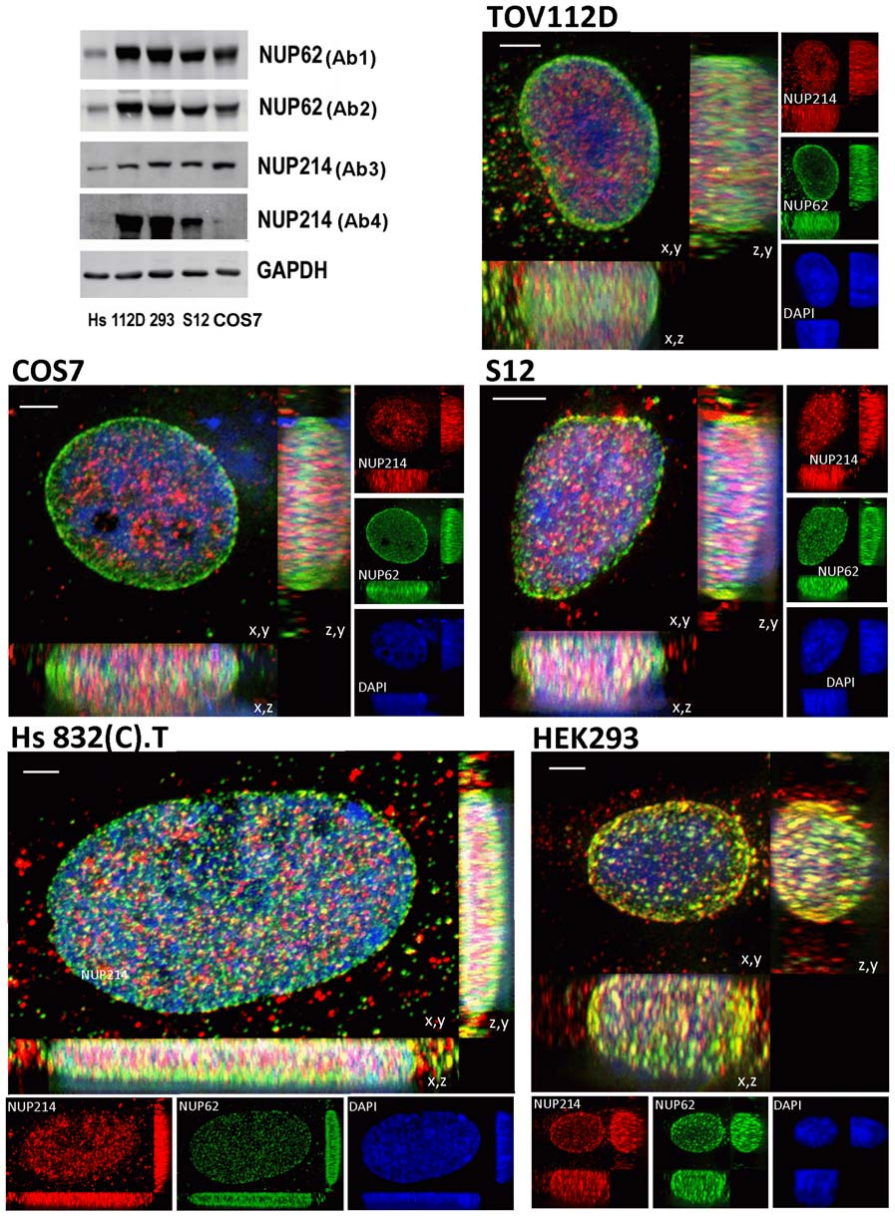
Total accumulation and distribution of NUP62 and NUP214 in different cell lines. Top left corner: immunoblot analyses of Hs 832(C).T (Hs), TOV112D (112D), HEK293 (293), S12, and COS7 (cos7) cell lysates. Antibodies are explained in the text. Immunofluorescent images for the indicated cell lines represent 3D deconvolved projections of 250 nm optical sections through 10 to 15 um of the z axis. Projections are shown on x,y, x,z, and z,y planes, as indicated. immunofluorescence analyses of NUP62 (green) and NUP214 (red) were generated using Ab1 and Ab4, respectively. Blue, DAPI stain; yellow, overlap between green and red. Bars represent 2 um.

Immunoblot analyses (Fig. 6) revealed varied total protein accumulation of both NUP62 and NUP214 in the following cultured cell lines: Hs 832(C).T, COS7, TOV112D, HEK293, and S12. Immunoblot analysis were performed with commercially available antibodies (see Materials and Methods) directed at distinct segments of residues on NUP62 (Ab1, human residues 24–178; Ab2, human residues 401–522) and two distinct segments of residues on NUP214 (Ab3, human residues 2050–2090; Ab4, human residues 1250–1300). For immunofluorescence analyses Ab1 and Ab4 were used as Ab2 and Ab3 did not produce discrete nuclear signals. The absence of a strong immunofluorescence signal for NUP62 Ab2 may result from steric interference, as the epitope(s) for this antibody lie within the coiled-coil domain of NUP62 that directly interacts with other nucleoporins [36]. The epitopes for NUP214 Ab4 and Ab3 lie within the N-terminal border and C-terminal regions of the FG-repeat containing domain, respectively [45]. Ab3 may not produce a signal in immunofluorescence analyses because the binding epitope(s) are obscured by a direct intermolecular interaction that occurs between the C-terminal extension (CTE) and β-propeller structures of NUP214 [45]. The immunoblot signals generated by NUP62 Ab1 and Ab2 were indistinguishable (Fig. 6). Although the immunoblot signals generated by NUP214 Ab3 and Ab4 were similar, difference in the intensity of NUP214 signals detected in Hs 832(C).T and COS7 cells suggests that post-translation modification may reduce binding of Ab4 in Western blots of lysates from these cell lines.

The relative abundance and the distribution of NUP62^+^/NUP214^−^, NUP62^−^/NUP214^+^, and NUP62^+^/NUP214^+^ immunolabeled NPCs varied between cultured cell types. Ovarian carcinoma TOV112D cells and COS7 cells displayed two major populations of NPCs: peripheral rim NUP62^+^/NUP214^−^ and contralateral surface NUP62^−^/NUP214^+^ NPCs, with few NUP62^+^/NUP214^+^ NPCs observed (Fig. 6). In HEK293 cells, NUP62^+^/NUP214^+^ NPCs predominated on the rim and contralateral surfaces, with some NUP62^−^/NUP214^+^ NPCs dispersed across the latter (Fig. 6). A human neuroblastoma cell line (S12) displayed NUP62^+^/NUP214^−^, NUP62^−^/NUP214^+^, and NUP62^+^/NUP214^+^ NPCs, the latter appearing mostly in segments near the periphery of the nucleus (Fig. 6). The Hs 832(C).T cells, which appear fibroblastoid, displayed few NUP62^+^/NUP214^+^ NPCs, with NUP62^+^/NUP214^−^ NPCs predominating and fewer NUP62^−^/NUP214^+^ NPCs on the contralateral nuclear surfaces (Fig. 6). Image stack projections also were viewed from the x,z and z,y planes, and differences in the distribution of NPC populations observed from these perspectives were consistent with those described above. In TOV112D cells, NUP62^+^/NUP214^−^ NPCs form an intense belt around the peripheral rim of the nucleus (Fig. 6; x,z and z,y planes). The NUP62^+^/NUP214^−^ NPC belt also is apparent at the nuclear rim of COS7 cells, and in HEK293 cells it appears to be populated by NUP62^+^/NUP214^+^ NPCs (Fig. 6; x,z and z,y planes). In neuroblastoma (S12) and benign human fibroblastoid (Hs 832(C).T) cells, NUP62^+^/NUP214^−^ NPCs appear from two regions of intense immunolabeling on the contralateral flattened surfaces of the nucleus (Fig. 6; x,z and z,y planes). As will be shown below, the intense belts of NUP62 immunolabeling on the nuclear rim observed in TOV112D and COS7 are not evident in S12 and Hs 832(C).T cells, replaced instead by relatively uniform NUP62 immunolabeling of NPCs over the surface of the nucleus.

**Figure 7:**
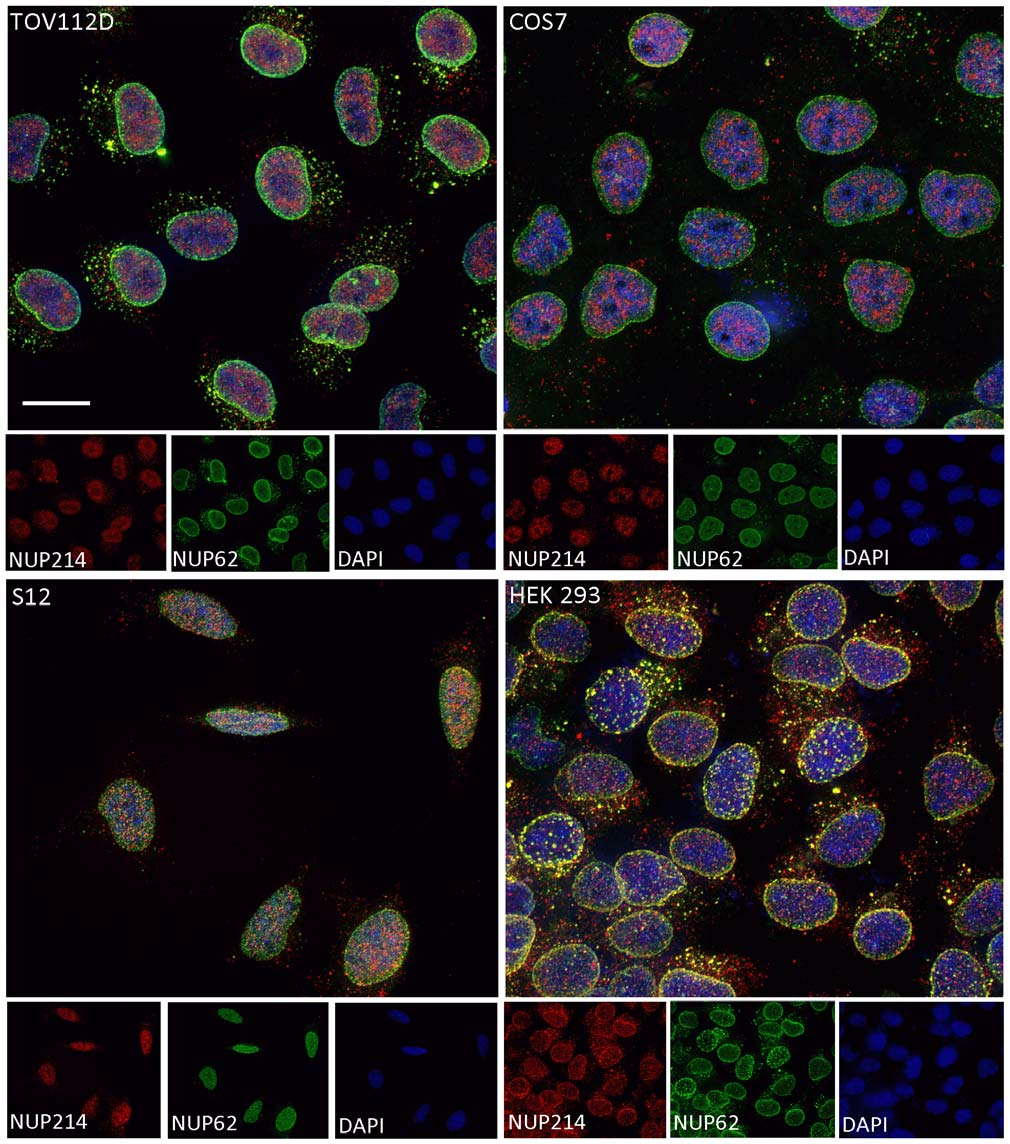
Nuclear distribution of NPC populations varies with cell type. Double immunofluorescence analyses of NUP62 (green) and NUP214 (red) are shown for large fields of subconfluent TOV112D ovarian carcinoma, COS7, S12 neuroblastoma, and HEK293 cells. Blue, DAPI stain; yellow, overlap between green and red. Bar represent 10 um.

**Figure 8:**
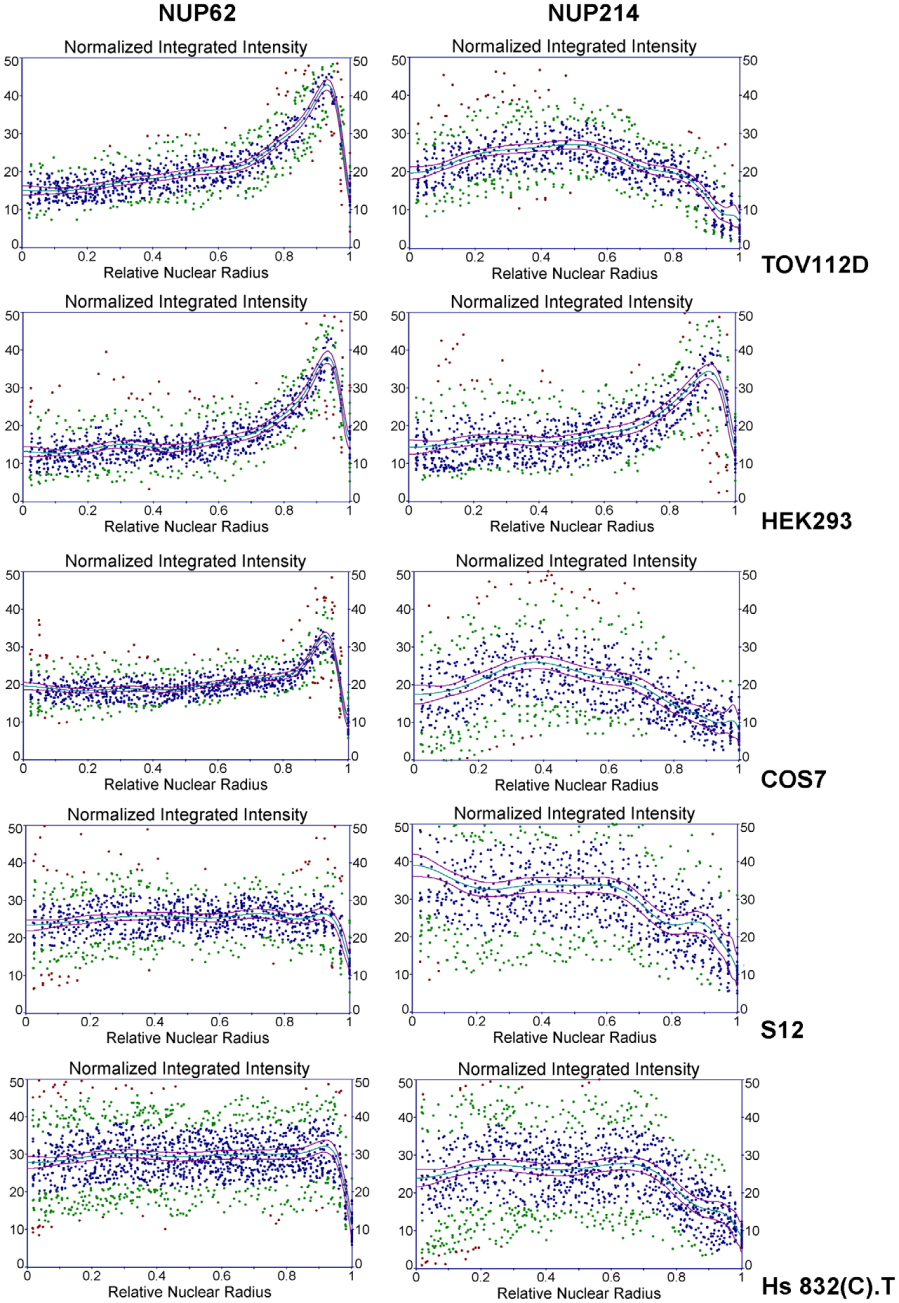
Radial intensity distribution graphs of NUP62 and NUP214 immunofluorescence in cultured cell lines. Immunofluorescent images for fields of TOV112D, HEK293 (293), COS7, or S12 cells (similar to those shown in Fig. 5), and images from several different Hs 832(C).T cells, were analyzed. Radial intensity distributions for NUP62 or NUP214 were determined for each cell line and normalized to arc length, and a minimum of 24 sectors derived from at least six independent nuclei were used for each analysis. Radial distances were normalized from 0 (point of origin) to 1 (surface of nuclear envelope). Distribution points are colored according to residuals (blue: less than one standard error; green: less than two standard errors; red: greater than two standard errors). Plotted in red are 95% confidence intervals.

As HEK293 cells displayed a large fraction of NPCs immunolabeled with both NUP62 and NUP214 antibodies (NUP62^+^/NUP214^+^ immunolabeled NPCs), binding of both NUP62 and NUP214 antibodies to the same NPC is not precluded if both nucleoporins are present. This supports the interpretation that differences in immunolabeling represent differences in the distribution of NUP62 and NUP214 among NPCs rather than variations in the accessibility of the epitopes. The observed NPC patterns of the cultured cell types were consistent among most of the cells in a proliferating culture (Fig. 7). Among the cells within each culture, overt changes in NPC patterning were only apparent in cells during M phase of the cell cycle. Images of the nuclei of several interphase cells were analyzed to determine radial distributions of NUP62 and NUP214 signals over multiple sectors. Integrated intensities for radial projections were normalized according to length, plotted against relative nuclear radial distance, and curves were fitted by polynomial regression. As shown in Fig. 8, differences between NUP62 and NUP214 nuclear immunolabeling patterns are readily apparent in TOV112D and COS7 cells. In addition, the analyses contrasts the NUP62 nuclear immunolabeling patterns among the cell lines. Preferential accumulation of NUP62^+^ NPCs at the nuclear rim appears to vary in degree, from a highly polarized distribution at the nuclear rim in TOV112D cells to a relatively even distribution in S12 and Hs 832(C).T cells (Fig. 8). In HEK293 cells, polarized distribution of both NUP62 and NUP214 is observed.

### High resolution immunofluorescence microscopy reveals architectural microheterogeneity in NPC populations

**Figure 9:**
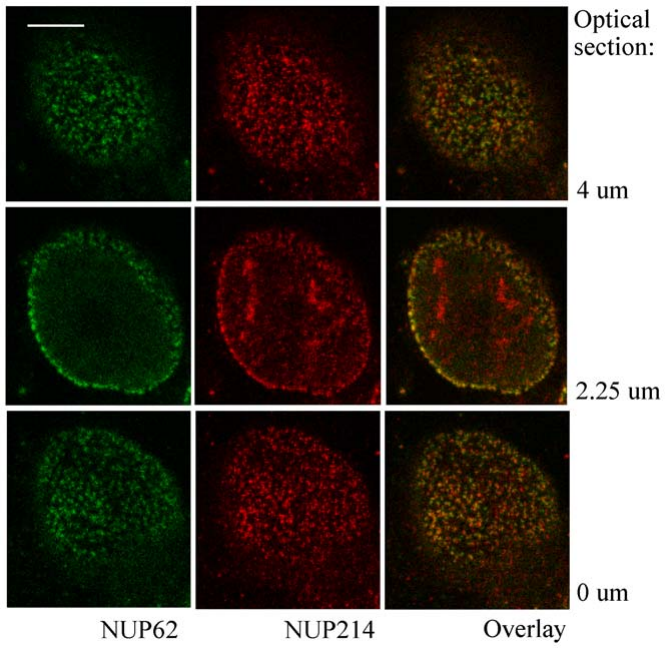
Optical sections of NUP62 and NUP214 immunolabeling in S12 cell nuclei using confocal and high resolution STED microscopy. HIgh resolution STED (NUP214; red) and standard confocal (NUP62; green) double immunofluorescence microscopy were performed on S12 neuroblastoma cells. Individual optical sections were selected from the ventral (culture plate side) nuclear surface (0 um) upward to the dorsal surface at intervals of 250 nm. Yellow, overlap bewteen green and red. Bar represents 5 um.

Stimulated emission depletion (STED, Leica) combined with scanning confocal microscopy resolved immunofluorescent images of individual NPCs. The S12 neuroblastoma cell line was chosen for this analysis as it displays each of the three permutations of NUP62/NUP214 immunolabeled NPC types. Individual optical sections from the surfaces facing the culture plate or the medium and the central plane of the S12 nucleus revealed both common and disparate immunolabeled patterns for NUP62 and NUP214 (Fig. 9). Punctate immunolabeling for NUP62 and NUP214 was revealed on the surfaces of the S12 nucleus, with partial overlap. The central plane revealed partial overlap of NUP62 and NUP214 immunolabeling on the nuclear rim. Intranuclear filiform accumulations of NUP214 also were detected (Fig. 9).

**Figure 10:**
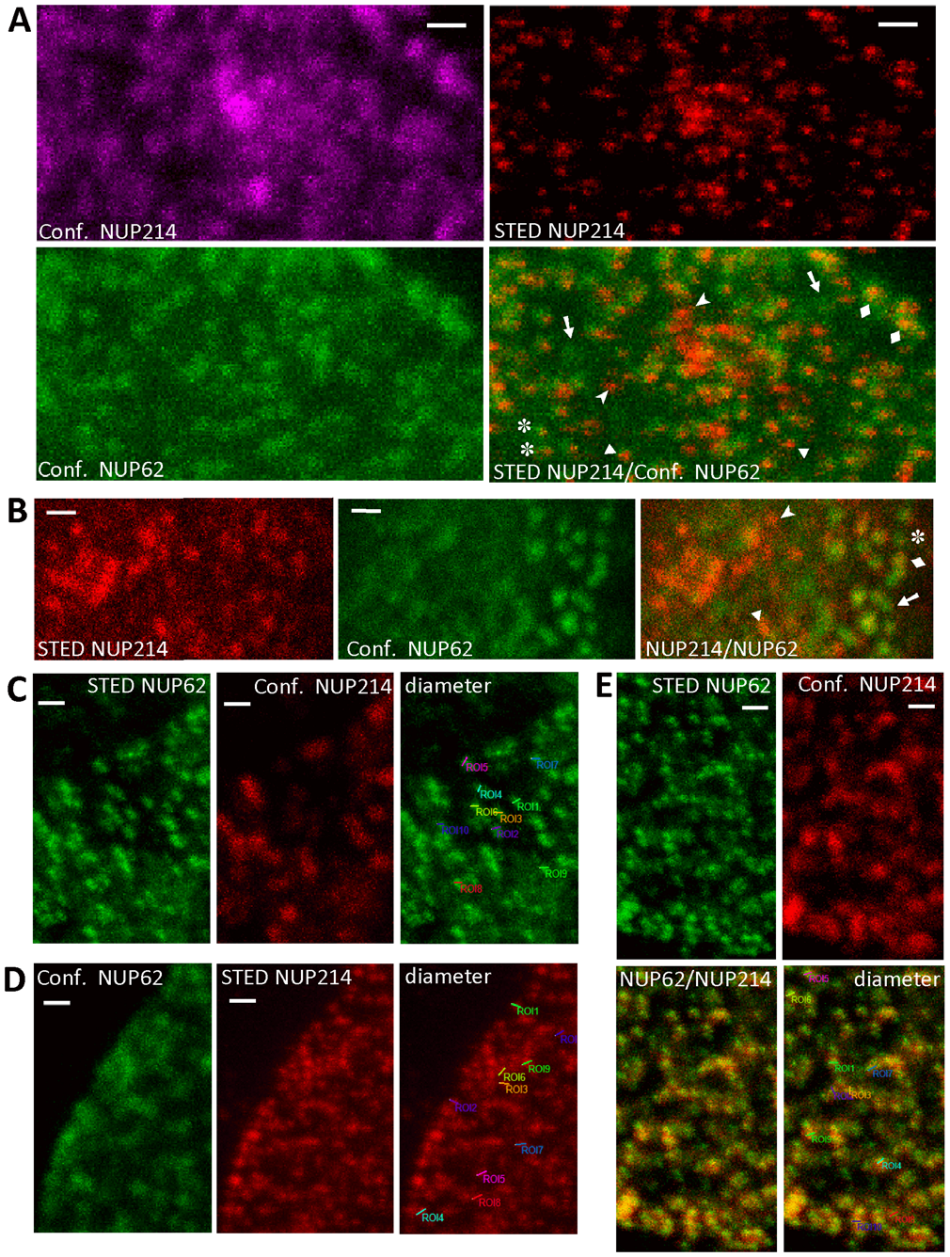
High resolution STED microscopy resolves NPC clusters, reveals microheterogeneity in NPC populations, and demonstrates that NPCs from different populations are similar in size. HIgh resolution STED microscopy (STED) and confocal (Conf.) double immunofluorescence microscopy were performed with NUP62 (green) and NUP214 (red) antibodies in S12 neuroblastoma cells. All images are projections of 8–10 um total optical z-stacks. Bars represent 500 nm. A. Comparison of confocal and STED microscopy resolution of NUP214 antibody immunofluorescence. A and B. Arrows, NUP62^+^/NUP214^−^ NPCs; asterisks, NUP62>NUP214 NPCs; diamonds, NUP62^+^/NUP214^+^ NPCs; triangles, NUP62<NUP214s; arrowheads, NUP62^−^/NUP214^+^ NPCs. C, D, and E. Measurement of diameters of NUP62^+^/NUP214^−^ (C), NUP62^−^/NUP214^+^ (D), and NUP62^+^/NUP214^+^ (E) NPCs in S12 cells. Measured pores were derived from STED microscopy images (indicated by asterisks). Yellow represents overlap between green and red.

The resolving power of STED microscopy allowed groups of individual NPCs to be distinguished from what appeared in standard confocal images to be large indefinite clusters (Fig. 10A). Microheterogeneity in NPC populations was revealed by STED microscopy: in addition to NUP62^+^/NUP214^−^ and NUP62^−^/NUP214^+^ NPC populations, NUP62>NUP214 and NUP62<NUP214 populations also were detected (Fig. 10A and 10B). Remarkably, STED microscopy revealed NUP214 as a single dot, consistent with a position on the cytoplasmic annular ring, whereas NUP62 was revealed as pairs of dots (the dots are inclined consistently, and the distance between the dots appears greater when viewed more laterally though NPCs nearer the rim), consistent with the NUP62 complex forming two rings in each pore (Fig. 10C and 10E). Finally, sufficient resolution was provided by STED to roughly compare the diameters of the NPC populations (derived from the STED images of NPCs shown in Fig. 10C, 10D, and 10E). Measured from each outer edge across the pore, NUP62^+^/NUP214^−^ NPCs measured 158±19 nm, NUP62^−^/NUP214^+^ NPCs measured 186±11 nm, and NUP62^+^/NUP214^+^ NPCs measured by the NUP62 antibody immunofluorescence as 178±11 nm. These values are similar, and suggest the absence of profound gross structural differences between the NPC populations. The size of isolated NPCs has been reported as 125 nm [46], differing from the observed values because of the physical contributions of primary and secondary antibodies and light diffraction.

## Discussion

Architectural diversity among NPCs could result from several mechanisms, including differential expression or distribution of nucleoporins, posttranslational modification of NPC proteins, and structural alterations associated with nucleocytoplasmic transport. Since many nucleoporins are distributed between NPCs and cytoplasmic and intranuclear cellular pools (discussed in [47]), differences in total expression may not manifest directly as differences in NPC composition. Similarly, cells with equivalent expression of a specific nucleoporin may not display equivalent distribution of that nucleoporin among NPCs, as allocation to cytoplasmic and intranuclear compartments may differ by cell type and directly impact distribution to NPCs. Posttranslation modifications of NUP62 and NUP214 include addition of O-linked N-acetylglucosamine [17], [48], [49], [50] and phosphorylation [51], [52], [53], and may affect nucleoporin immunolabeling patterns if present on relevant epitopes in specific populations of NPCs. Alterations in NUP214 structure associated with its status in nucleocytoplasmic transport processes [32] may also affect immunolabeling.

The results presented here suggest that cell-type associated differences in NUP62 and NUP214 NPC immunolabeling patterns arise from sorting of these FG-repeat nucleoporins to specific NPC populations. The co-localization of NUP62 and NUP214 in some NPCs indicates that binding of one nucleoporin antibody does not sterically preclude binding of the other to the same NPC, while studies of epitope-tagged NUP62 and NUP214 supported the conclusion that differences the immunolabeling patterns observed using native antibodies resulted from the presence or absence of nucleoporins and not from post-translation modifications affecting antibody binding. Immunoblot analyses suggest that posttranslational modifications may attenuate binding of the NUP214 antibody in Western blot applications, but this putative modification does not appear to affect the pattern of NPC immunolabeling observed with this antibody in immunofluorescence applications. Sorting of NUP62 and NUP214 to different NPC populations does not conflict with other data showing that NUP62 and NUP214 can physically associate [36]. Physical association of NUP62 and NUP214 has been observed in cell lysates and using recombinant proteins in vitro, and such studies do not directly address the distribution of the NUP62/NUP214 association in vivo. Clearly, complexes of NUP62 and NUP214 may form in those NPCs which contain both nucleoporins. In addition, recombinant NUP62 bound a form of NUP214 from cell lysates that migrated slower during SDS-PAGE than the form(s) that bound other nucleoporins, such as NUP153 [36]. Immunoblot analyses presented here also suggest the presence of slower and faster migrating forms of NUP214, and future studies may reveal a correlation between the expression or accumulation of NUP214 forms and the distribution of NUP62 and NUP214 among NPC populations in different cell types.

Several studies have shown that distribution of NPCs reflects the transcriptional disposition of the underlying chromatin [54]. In general, DNA in the vicinity of the nuclear envelope in metazoans appears heterochromatic and to contain silent genes [55]. Studies in *C. elegans* have indicated that during cellular differentiation, induction of transcription is accompanied by gene migration from the periphery to central regions of the nucleus [56]. Consistent with these findings are studies in Drosophila salivary and embryonic cells showing that a large set of genes involved in cell cycle regulation and development make contact with FG-containing nucleoporins in the nucleoplasm at sites distal to the NPC. As determined by histone H3 methylation, histone H4 acetylation, and other criteria, these genes on average are transcriptionally more active than genes associated with NPCs [30], [31]. We have observed intranuclear accumulation of protein that binds NUP214 antibody, although this immunolabeling was relatively resistant to siRNA-mediated knockdown. It is possible that intranuclear NUP214 immunolabeling occurs in regions of active transcription, as the labeling appears to occur in areas of less intense DAPI staining (characteristic of euchromatin).

Chromatin associated with NPCs has been found to be enriched in a subset of genes involved in development and cell cycle regulation, albeit in a transcriptionally less active state than genes associated with nucleoplasmic nucleoporins [30]. From those studies, activation of silent genes at the nuclear periphery by transcriptional regulators appears to result in inward migration either accompanied by or followed by association with nucleoplasmic nucleoporins. Thus, while actively transcribing genes may be transferred to the inner nucleus, genes poised to receive positive regulators for activation may be retained near or moved to the nuclear envelope. One of the functions of the FG-repeat proteins is to direct specific cargo through the NPC, and NPCs bearing different FG-repeat proteins would be expected to transport different cargo. In addition, the specific functions of FG-repeat proteins in nuclear transport differ, and may even be antagonist [29]. The NUP214-NUP88 complex is required for NF-κB translocation into the nucleus [57], [58], indicating that NUP214^+^ NPCs mediate this process. The characteristic distribution of NUP214^+^ NPCs suggests that NF-βκB is given access to specific subnuclear domains, and consequently to specific genes. Alternatively, the MUC1-C oncoprotein directly binds the central domain of NUP62, and this interaction is required both for nuclear import and for the oncogenic manifestations of MUC1-C over-expression in carcinoma cells [59]. The activity of MUC1-C would be directed at subnuclear domains accessed by NUP62^+^ NPCs. Similarly, nuclear delivery of the glucocorticoid receptor complex would be mediated by NUP62^+^ NPCs [60]. Targeting to specific subnuclear domains, and hence to specific genes, could be conferred by cell-type specific expression and sorting of FG-repeat proteins among NPC populations. Modulation of the distribution and composition of NPC populations may constitutes an epigenetic mechanism through which cell type-specific patterns of gene expression can be generated from more general external and intrinsic signaling streams. Recruitment of target genes to the nuclear periphery and to the domains of specific NPCs thus may program how the cell responds to future cues [61].

As nuclear exits, NPCs control the first stage of delivering nascent RNAs to specific cytoplasmic sectors, and thereby may play an important role in controlling subcellular organization. The identification and mapping of NPC populations divulges a novel parameter for illuminating nuclear polarity, which through the selective delivery of RNAs could contribute to or be required for the development and/or maintenance of cellular polarity. Transport of RNA from nuclear to cytoplasmic domains requires certain FG-repeat proteins [28], and some evidence has suggested that different RNAs may require specific FG-repeat proteins for nuclear export [62]. Variations in the distribution of NPC populations reported here suggest that transcripts may be selectively delivered to specific cytoplasmic sectors in certain cell types, thereby mediating differentiation and the acquisition of polarity. Understanding NPC diversity and distribution will help elucidate how the 3D structure of the nucleus contributes to cell asymmetry and differentiation, as well as to patterning and stereological control of gene expression.

## Materials and Methods

### Cell lines and antibodies

Human ovarian carcinoma (TOV112D and TOV21G), benign cystadenoma cells (Hs 832(C).T), COS7, and HEK293 cell lines were either purchased from the American Type Culture Collection (ATCC) or acquired from long-term laboratory stocks. The S12 neuroblastoma cell line is a spontaneously differentiating clone derived from SH-SY5Y [63]. All cells were maintained Dulbecco's modified Eagle medium (DMEM; Cellgro) supplemented with 15% fetal bovine serum. Antibodies to NUP62 were purchased from BD Transduction Laboratories (product numbers 611962 and 610498 (Ab1)) and Santa Cruz Biotechnology (product number H-122 sc-25523 (Ab2)). Antibodies to NUP214 were purchased from Bethyl Laboratories (product numbers IHC-00103 (Ab4) and A300–717A (Ab3)). Antibody to NUP133 was purchased from Abnova (product number H00055746-M01). Antibody to GAPDH (MAB374) was purchased from Chemicon (Millipore). Monoclonal anti-V5 epitope antibody was purchased from Thermo Scientific (product number MA1-81617). All secondary antibodies were purchased from Jackson ImmunoResearch Laboratories.

### Immunofluorescence microscopy

Subconfluent cells were cultured on glass coverslips. Immunofluorescence staining was performed similarly to Maeshima et.al [64]. Cells were fixed with 3.7% paraformaldehyde in PBS and quenched with 50 mM glycine in HMK buffer (20 mM HEPES; pH 7.5, 1 mM MgCl_2_, 100 mM KCl). Coverslips were washed with HMK briefly. After cells were treated with 0.5% Triton X-100 in HMK, they were treated with blocking solution (10% normal donkey serum and 1% BSA in HMK buffer). Cells were incubated with primary antibody for 1 to 2 hours at room temperature. After washing with HMK five times, the cells were incubated with secondary antibodies (Jackson ImmunoResearch). Cells were washed in PBS and mounted with VectaShield mounting medium with DAPI (Vector Laboratories). For double immunofluorescence for NUP62 and NUP133, cells were first incubated with NUP133 antibody, then washed and incubated with anti-mouse secondary antibody (Cy5), then washed and incubated with FITC-coupled NUP62 antibody (45 min).

Epifluorescence microscopy was performed using a Zeiss Axioplan 2 microscope. Images were acquired with either 40× (air) or 63× (oil) objectives in z-stack series extending 10 to 15 um. Images were analyzed after 2D or 3D deconvolution (AutoQuant X; MediaCybernetics) and image stacks were projected on single planes. Confocal microscope was performed with the Leica TCS SP5 microscope, using the 488 nm and 543 nm lines of the conventional lasers. Stimulated emission depletion (STED) microscopy was performed with the Leica TCS STED microscope, using the 770 nm wavelength for depletion and the 635 nm pulse laser for excitation. All confocal and STED images were take using a 100×1.4NA objective (oil). Images for confocal and STED microscopy were acquired in 8 to 10 um Z-stacks, and projected without deconvolution.

Radial intensity distributions for nuclear sectors were defined and quantified using the Radial Profile Extended plug-in for Image J (Philippe Carl; University of Munster, Germany). Region of interest radii were set to extend from the local center of the nucleus to its boundary with the cytoplasm; radial extension integration angle was set at 20° (40° total), and intensity data was normalized to the length of each radial projection. Curve fitting by polynomial regression analysis and determination of confidence intervals was performed using TableCurve version 5.01 (Systat Software). All curves were fitted by polynomial regression to y = a+bx^2^+cx^4^+dx^6^+ex^8^+fx^10^+gx^12^+hx^14^+ix^16^+jx^18^+kx^20^.

### Immunoblotting

Cell lysates were prepared by washing cells once in PBS followed by resuspension in SDS sample buffer (62.5 mM Tris-HCl, pH 6.8, 10% Glycerol, 2% SDS, 5% β-mercaptoethanol, 125 ug/ml bromophenol blue). Samples were heated to 90° for five minutes, cooled, and resolved by SDS-PAGE. Proteins were transferred to nitrocellulose sheets electrophoretically in transfer buffer (25 mM Tris, 92 mM glycine, 20% v/v methanol) and stored until use at −20°. Nuclear and cytosolic fractions were generated by the REAP method [43], which is rapid, minimizes exposure to hyperosmotic conditions, and preserves protein-protein intaractions. Briefly, cell monolayers are chilled on ice, scraped into PBS, and washed. One third of the samples is removed for the whole cell lysate. The samples are pelleted and lysed in an isotonic buffer containing 0.1% NP40, then pelleted. The supernatant is taken as the cytosolic fraction, the pellet is washed once in lysis buffer, and the washed pellet is dissolved in SDS sample buffer and used as the nuclear fraction. Samples were processed for SDS-PAGE as described above. Mmembranes were blocked for 2 hours at room temperature with 5% nonfat milk in TBS (25 mM Tris pH 7.4, 100 mM NaCl). The blots were then incubated at 4° overnight in blotting solution (TBS supplemented with 5% nonfat milk, 1–5% horse serum, 0.5% Tween 20) with primary antibody (0.5–2.5 ug/ml). The blots were washed three times, and incubated with secondary antibody at room temperature in TBS/Tween 20 for 1.5 hours. Immunoreactive bands were visualized using enhanced chemiluminescence (ECL, Amersham) or with the Amersham ECL Plex immunoblotting system (GE Healthcare). The latter were scanned by the Typhoon Trio (GE Healthcare).

### Knockdown by siRNA transfection

For siRNA knockdown experiments, cells were plated at 3×10^4^ per cm^2^ using 15% FBS in DMEM without addition of antibiotics for 24 hours. Cells were transfected 24 hours after plating with siRNA using Lipofectamine RNAiMAX (Invitrogen) and the manufacturers' recommended procedures. The siRNAs for NUP62 knockdown (12–24 pmol) were purchased from Santa Cruz Biotechnology (product number sc-36107), and cells were analyzed 48–72 hours after transfection. The siRNAs for NUP214 knockdown (75 pmol) comprised a mixture of three custom siRNAs (5′-GUGCAGGAAUUGAAGAAUAuu-3′, 5′-UCGACAAAGUGAUCAGAUUuu-3′, and 5′-GGGAGUUGUCGUAGACUAUuu-3′) combined with commercial siRNAs for NUP214 from Santa Cruz Biotechnology (product number sc-106320). Cells were incubated with the NUP214 siRNA mixture for 3 days and changed to new fresh media with new siRNAs for another 3 days before analysis.

### DNA-mediated transfections

Plasmids containing the cDNA clones for human NUP62 and NUP214 were obtained from Open Biosystems. The V5 epitope tag (GKPIPNPLLGLDST) plus a strong Kozak consensus sequence and start codon were inserted in frame in place of the methionine start codons for NUP62 and NUP214 (insert: GCCACCATGGCGGGCAAACCGATTCCGAACCCGCTGCTGGGCCTGGATAGCACC). The modified cDNAs were directionally cloned into the Not I and Sal 1 sites of pCMV-Sport-6 (Invitrogen). Cells were transfected using either Lipo293D (SignaGen Laboratories), FuGENE HD (Promega), or Lipofectamine (Invitrogen) according to the instructions provided by the manufacturers.
